# Dental Anomalies in Saudi Arabia: A Systematic Review

**DOI:** 10.3390/healthcare12232323

**Published:** 2024-11-21

**Authors:** Khalid Aljohani, Hanan Shanab, Ali Alqarni, Khalid Merdad

**Affiliations:** 1 Department of Oral Diagnostic Sciences, Faculty of Dentistry, King Abdulaziz University, Jeddah 21589, Saudi Arabia; 2 Department of Oral and Maxillofacial Surgery and Diagnostic Sciences, College of Dentistry, Majmaah University, Majmaah 11952, Saudi Arabia; h.hamzih@mu.edu.sa; 3 Department of Oral & Maxillofacial Surgery and Diagnostic Sciences, Faculty of Dentistry, Taif University, Taif 21944, Saudi Arabia; aqarni@tu.edu.sa; 4 Department of Endodontics, Faculty of Dentistry, King Abdulaziz University, Jeddah 21589, Saudi Arabia; kmerdad@kau.edu.sa

**Keywords:** dental anomalies, congenitally missing, supernumerary, ectopic eruption, rotation

## Abstract

Background: Epidemiological studies have shown varying prevalence rates of dental anomalies worldwide, ranging from 5.2% to 56.9%, with a higher rate of 90.4% in patients with cleft lip and palate. In Saudi Arabia, studies have also reported varied prevalence rates, likely due to genetic differences or sampling variations. However, no research has yet evaluated the quality of these studies or provided an overall prevalence estimate, which is the aim of the present study. This systematic review aims to assess the prevalence and types of dental anomalies across various regions of the Kingdom of Saudi Arabia (KSA). Methods: A comprehensive literature search identified 10 relevant studies on different dental anomalies in Saudi Arabia. The quality of the enrolled studies was assessed using the Newcastle–Ottawa Scale (NOS), showing variability in the methodological quality of the included cohort studies, with several studies demonstrating a moderate to high risk of bias. Results: Common anomalies included hypodontia, hyperdontia, microdontia, and impacted teeth. This study highlights the varying prevalence of dental anomalies in different regions of Saudi Arabia, ranging from 2.6% to 45.1%. Conclusions: This review highlights the need for early diagnosis and tailored treatment approaches to mitigate the clinical challenges posed by these anomalies, underscoring the importance of standardized diagnostic criteria and further research to understand regional and demographic differences in the prevalence of dental anomalies in Saudi Arabia.

## 1. Introduction

Dental anomalies are congenital abnormalities that can develop alone or as part of a syndrome [1,2,3]; they are often detected clinically or through radiographic examinations during the mixed dentition stage, which begins around the age of 6 [3,4,5,6]. Early diagnosis can aid in improving occlusion and planning orthodontic treatment [7]. The etiology behind these anomalies is not fully understood but is believed to be multifactorial and mainly caused by genetic, epigenetic, and/or environmental factors [8,9].

Dental anomalies may affect the aesthetics and, consequently, the quality of life of the patient, and the management of these conditions through different dental procedures can thus improve the oral health, psychological wellbeing, and quality of life of the patient [10,11]. These conditions can include abnormalities in the number, size, form, and/or structure of teeth, and they are included in the International Classification of Disease version 10 among disorders of tooth development and eruption (diseases of the oral cavity, salivary glands, and jaw) (WHO-ICD-10; 2019) (K00.2-K14) [12].

Certain anomalies, such as supernumerary roots, invaginated teeth, and taurodontism, do not significantly affect oral health but may necessitate more attention and specialist management during dental treatments such as root canal treatment, extraction, or orthodontic treatment [13,14,15]. However, anomalies such as congenitally missing teeth or teeth with enamel defects pose clinical challenges, causing developmental abnormalities and malocclusions that require early detection and proper treatment planning [16]. Early recognition and proper management are crucial to ensuring better dental development and providing good dental aesthetics and function [17]. 

Many epidemiological studies have reported the prevalence of dental anomalies across different geographical regions worldwide [18,19,20,21,22,23,24,25,26,27]. There is wide variation in their prevalence, which may reflect ethnic differences in these conditions [18,19,20,21,22,23,24,25,26,27]. The prevalence ranges from 5.2% to 56.9%, and it was found to be higher among patients with cleft lip and palate (90.4%) [22].

Several studies have reported the prevalence of dental anomalies in various cities and regions in the Kingdom of Saudi Arabia (KSA) [1,3,4,28,29,30,31,32,33]. Variations in the results of these studies could be attributed to genetic differences in the population or variations in the sampling process [8,9]. However, to the best of our knowledge, no study has assessed the quality of these studies and provided an overall prevalence estimate. Consequently, the aim of the present study is to determine the prevalence and types of dental anomalies in different regions of the Kingdom of Saudi Arabia.

## 2. Materials and Methods

The protocol for this systematic review was developed following the PRISMA (Preferred Reporting Items for Systematic Reviews and Meta-Analyses) statement and followed the methodology of other systematic reviews [34]. This review was conducted by an investigative team that included a lead reviewer and two independent reviewers (A.A. and K.A.). The process was designed to address the following focused question:

What is the prevalence of dental anomalies in Saudi Arabia?

As part of the PRISMA requirements, we formulated the following PECO criteria:

Population: Individuals residing in the Kingdom of Saudi Arabia (KSA).

Exposure: Presence of dental anomalies, as identified through observational studies conducted within the KSA.

Comparison: General population or individuals without diagnosed dental anomalies (if applicable based on study design).

Outcomes: Prevalence and types of dental anomalies reported, along with diagnostic criteria and methods used.

### 2.1. Information Sources and Search Strategy

A comprehensive search strategy was developed to identify relevant studies on the prevalence of dental anomalies in Saudi Arabia. Searches were conducted on the electronic databases PubMed, EMBASE, OVID, and Web of Science up to October 2024. The initial search was supplemented by examining the reference sections of the identified articles to capture any additional relevant studies. Additionally, a manual search was performed in the latest issues of prominent Saudi dental journals, including *The Saudi Dental Journal*, *The Journal of Contemporary Dental Practice*, *The Saudi Journal of Oral Sciences*, and *The Saudi Journal for Dental Research*. The search strategy included detailed keywords such as ((dental) AND (anomalies) OR anomaly) AND (Saudi Arabia).

### 2.2. Eligibility Criteria

Studies were eligible for inclusion if they were observational studies conducted in the Kingdom of Saudi Arabia (KSA) and were published in Arabic or English up to October 2024.

Studies focusing on soft tissue abnormalities, reviews, commentaries, case reports, and editorials were excluded.

### 2.3. Study Selection and Data Collection

All articles that met the inclusion criteria, based on their titles and abstracts, were collected. Two independent reviewers (A.A. and K.A.) reviewed the titles and abstracts of the initial list of potentially relevant articles. Prior to data extraction, a calibration process was conducted to ensure consistency in data selection and extraction. The reviewers initially evaluated a 20% sample of the studies to achieve inter-examiner agreement (kappa ≥ 0.81). Following calibration, the reviewers independently completed the screening process for eligible studies.

Data extraction was then conducted independently by the two reviewers. For each included study, the following information was recorded: authors and publication year, study design, sample size and demographics, prevalence and types of dental anomalies reported, and diagnostic criteria and methods. Any discrepancies during data extraction were resolved through discussion, with a third reviewer available for consultation if needed.

### 2.4. Risk of Bias Assessment

Two reviewers (A.A. and K.A.) independently assessed the risk of bias for each included study, using the Newcastle–Ottawa Scale (NOS) for quality assessment [35,36]. The NOS evaluates three quality parameters (selection, comparability, and outcome), with a maximum score of 9 points [35,36]. Studies scoring ≥ 7 on the NOS were considered to be high-quality studies, those with 7 or 6 points were considered to have a medium risk of bias, and those with 5 points or less were considered to have a high risk of bias [35,36]. With regard to selection, the study was considered to have a low, medium, or high risk of bias if it scored 4, 2–3, or 1 point, respectively [35,36]. With respect to comparability, the study was considered to have a low, medium, or high risk of bias if it scored 2, 1, or 0 points, respectively [35,36]. With respect to outcome, the study was considered to have a low, medium, or high risk of bias if it scored 3, 2, or 1 point, respectively. 

Any disagreements in the quality assessments were resolved through discussion between A.A. and K.A.

### 2.5. Ethical Considerations

Since this review involves previously published articles, ethical approval was not required. Nevertheless, principles of ethical research, such as transparency, accuracy, and integrity, were strictly followed throughout the review process.

## 3. Results

**Figure 1:**
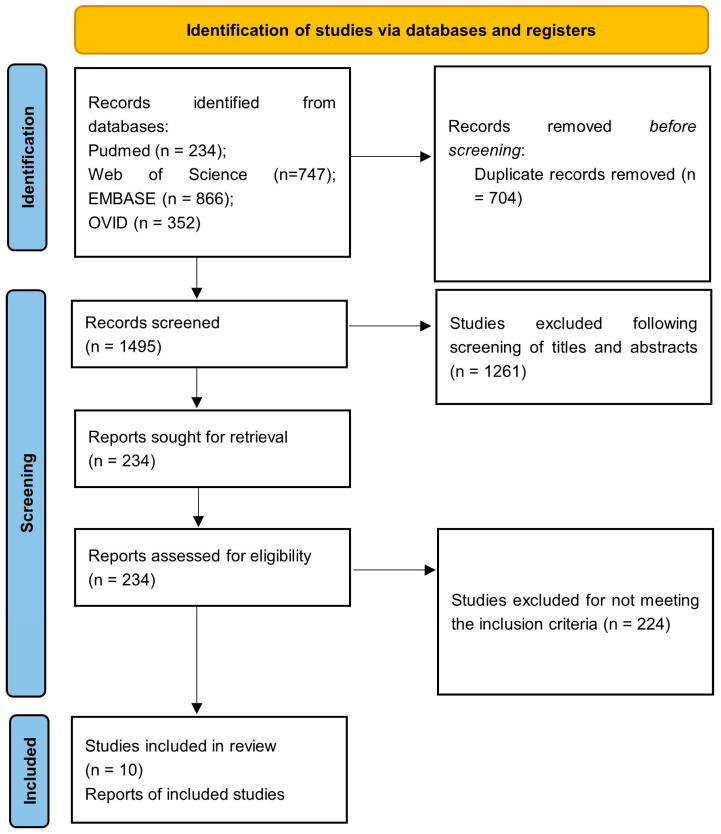
Reported published papers according to the PRISMA flowchart for the keywords used in the literature review.

This systematic review followed the Preferred Reporting Items for Systematic Reviews and Meta-Analyses (PRISMA) statement guidelines, along with the methodology of other systematic reviews; key aspects of the protocol are summarized below [37]. The supplementary PRISMA checklist can be found in Supplementary Table S1. Following the removal of duplicates and studies that did not meet the inclusion criteria, a total of 10 studies were included in the review (Figure 1) [34,37].

### 3.1. Risk of Bias Assessment

The risk of bias assessment for the cohort studies included in this review was conducted using the Newcastle–Ottawa Scale (NOS). The assessment focused on three main domains: selection, comparability, and outcome. The results are summarized in Table 1 [35]. With regard to the selection domain, the representativeness of the exposed cohort and the ascertainment of exposure were consistently rated positively across all studies. However, the selection of the non-exposed cohort and the presence of the outcome of interest at the start of the study were areas where most studies did not meet the criteria. Taking into account the comparability domain, none of the studies achieved a high score, indicating a general lack of control for confounding variables across the included studies. Finally, in terms of the outcome domain, the ascertainment of outcomes and the adequacy of follow-up were generally rated well; however, the duration of follow-up was often insufficient to observe the outcomes of interest. Overall, the studies by Ghaznawi et al. (1999) [4], Afify and Zawawi (2012) [3], and Vani et al. (2016) [28] each received a total score of 4, indicating a moderate risk of bias. The study by Yassin (2016) received a score of 3, while the remaining studies, including those by Aljuaid et al. (2022) [30], ALHumaid et al. (2021) [1], Bakhurji et al. (2021) [31], Qutub et al. (2021) [32], Renugalakshmi et al. (2023) [33], and Al-Jabaa and Aldrees (2013) [38], each received a score of 2, indicating a higher risk of bias.

### 3.2. Synthesis of Results

The included studies’ participants were from eleven different Saudi regions and cities, as follows: nine studies from Riyadh; six from Jeddah and Makkah; two studies from each part of the Eastern Province, Taif, Abha, and Jazan; and one study each from Najran, Sakaka, Qassim, and Alkharj. The publications included both males and females [1,3,4,28,29,30,31,32,33,38] (Table 2).

Most of the Saudi studies [3,28] examined patients with at least one dental anomaly (28.7–45.1%). Two or more dental anomalies were reported by Vani et al. (2016), Yassin (2016), ALHumaid et al. (2021), Aljuaid et al. (2021), and Renugalakshmi et al. (2023) [1,28,29,30,33]. The majority of the reported dental anomalies were present in the maxillary molar region, followed by the mandibular molar region, maxillary premolar region, mandibular premolar region, maxillary anterior region, and finally the mandibular anterior region [1]. Renugalakshmi et al. (2023) reported that anomalies were more common in the mandible than the maxilla, and were found in both jaws in 6.3% of participants [33]. Anomalies usually affected both sides (58.2%); they were reported on the right and left sides of the mouth in 24.68% and 17.09% of patients, respectively [33].

The reported anomalies can be classified according to the number, structure, shape, or position of the affected teeth (Table 3).

#### 3.2.1. Anomalies in Tooth Number (Missing Teeth/Hypodontia)

Congenitally missing teeth have been reported as one of the more common developmental anomalies of dentition [28,38]. In the KSA, their prevalence ranges from 5.4% to 31.68% [1,3,4,28,29,30,31,32,33,38]. 

Different studies reported a different order and prevalence of missing teeth. The most commonly missing teeth were mandibular second premolars [1,29,39]. In a study conducted in Jazan by Renugalakshmi et al. (2023) [33], 27.3% of missing teeth were left mandibular premolars. Other commonly reported missing teeth were maxillary permanent lateral incisors [29], maxillary permanent first premolars, and lateral incisors [1]. In a different study, the most commonly missing teeth were premolars, followed by lateral incisors and canines, which were more commonly positioned on the palatal side [40]. The least commonly missing teeth were central incisors, which were usually unilateral [32]. In primary dentition, the maxillary lateral incisor had the highest incidence of hypodontia (9%), as demonstrated in a study by Salama et al. (1994) [41].

Hypodontia is more common amongst females (20–41.57%) than males (19–20.00%) [33,38,42]; it is more prevalent in children under 10 years of age and generally affects the mandibular rather than the maxillary teeth, with the highest prevalence of 31.68% reported in Jazan by Renugalakshmi et al. (2023) [33]. It has been reported that hypodontia is more common in lateral incisors (4.0%) [43]. Patients usually present with multiple missing teeth. In contrast, oligodontia (anomalies associated with the congenital absence of six or more teeth, excluding the third molars) is rare (0.1–0.9%) and is more prevalent in females than males [2].

##### Supernumerary Teeth (Hyperdontia)

Supernumerary teeth, or hyperdontia, refer to the presence of one or more extra teeth in the maxilla or mandible [1,3,28,29,31,32]. Supernumerary teeth were reported in 0.3–3.0% of participants [1,3,28,29,31,32]. Hyperdontia is more common amongst males (4.3–7.2%) than females (1.2–4.0%) [32,33,38]. Most supernumerary teeth were found in the anterior maxilla (57.3%) and were single teeth, followed by double teeth (30.9%), and then multiple teeth (11.8%) [40].

#### 3.2.2. Anomalies in Tooth Shape

##### Macrodontia

Macrodontia is a rare anomaly where teeth are abnormally large [44]; it was reported in 0.3–2.1% of participants [3,29,30,33]. In a study conducted by Alyami et al. (2019), macrodontia was more common in maxillary incisors [40].

##### Microdontia

Microdontia is a condition where teeth are abnormally small [45]. Microdontia is more prevalent in the KSA compared to macrodontia, with a range of 2.4–12.6% [1,3,28,29,33]. A study by Alyami et al. (2019) reported that microdontia was more common in maxillary lateral incisors [40].

##### Fused Teeth

Fusion is the union of two normally separated tooth germs, resulting in a single large tooth [40]. The reported prevalence of fusion in the KSA ranged from 0.3% to 2.3% [3,28,29,33]. Fusion was more common in mandibular teeth [40].

##### Gemination

Gemination is a developmental anomaly in which a single tooth germ attempts to divide, resulting in a single large tooth with a bifid crown [46,47]. The reported prevalence of gemination in the KSA ranged from 0.3% to 2.4% [3,28,29,33]. Gemination was more common in mandibular incisors [40].

#### 3.2.3. Anomalies in Tooth Structure

##### Dens Invaginatus

Dens invaginatus, also known as dens in dente, is a dental anomaly resulting from the invagination of the enamel organ into the dental papilla during tooth development [48]. The reported prevalence of dens invaginatus in the KSA ranged from 0.3% to 2.7% [3,28,29,33]. Dens invaginatus was more common in maxillary lateral incisors [40].

##### Dens Evaginatus

Dens evaginatus is a dental anomaly characterized by the presence of an extra cusp or tubercle in the occlusal surface of the tooth [49]. The reported prevalence of dens evaginatus in the KSA ranged from 0.3% to 1.5% [3,29,33]. Dens evaginatus was more common in premolars [40].

##### Dilaceration

Dilaceration is a dental anomaly characterized by a curve in the root or crown of a tooth [50]. The reported prevalence of dilaceration in the KSA ranged from 0.3% to 4.5% [3,29,33]. Dilaceration was more common in maxillary central incisors [40].

##### Taurodontism

Taurodontism is a dental anomaly where teeth have an enlarged pulp chamber and shortened roots [51]. Studies reported the prevalence of taurodontism in the KSA to range from 0.3% to 5.2% [3,29,33]. Taurodontism was more common in molars [40].

#### 3.2.4. Anomalies in Tooth Position

##### Transposition

Tooth transposition is defined as when two adjacent teeth have exchanged positions [51]. The reported prevalence of tooth transposition in the KSA ranged from 0.3% to 0.9% [3,29,33]. Tooth transposition was more common in the canine and first premolar regions [40].

##### Impacted Teeth

Impaction is a condition where a tooth fails to erupt into the dental arch within the expected developmental window [52]. The reported prevalence of impacted teeth in the KSA ranged from 1.1% to 19.4% [3,28,29,33]. Impacted teeth were more common in the maxillary canine region [40].

#### 3.2.5. Other Dental Anomalies

##### Root Dilacerations

Root dilaceration was reported to be in the range of 1.14–7.8% [3,4,28,30]. This was the third most common anomaly reported by Bawazeer et al. in 2019 (7.1%) [43]; additionally, it was the most common anomaly observed in the AlKharj population [16], Eastern Region (Dammam) [1], and Jazan [33]. 

In 2023, Renugalakshmi et al. reported the highest incidence of root dilacerations (47.83%) [33]. This condition was more common amongst male patients (59.15%) than females (38.2%), and it affected the mandibular teeth more commonly than the maxillary teeth; it was also observed to be more common amongst children over the age of 10 years [33]. In another study [1], the most commonly reported dilaceration was in the mandibular third molars, followed by the mandibular second molars, maxillary second premolars, and mandibular incisors. The dental anomalies reported in the KSA are summarized according to their published paper (Table 2) and to the type of dental anomaly (Table 3).

## 4. Discussion

This study provides a pioneering comprehensive review of the prevalence and types of dental anomalies reported in different regions of the Kingdom of Saudi Arabia [1,3,4,28,29,30,31,32,33,38]. 

There are a number of limitations affecting the results of this study. Most of the reviewed studies were from a single center, which could have been affected by selection bias. Furthermore, the vast majority of the studies were of a retrospective design, with all of the limitations associated with this type of study, such as selection bias and missing information. The quality of the studies varied, with some studies lacking robust control groups or blinding, increasing their risk of bias. Additionally, there was a lack of standardization across the studies in terms of diagnostic criteria, methodologies, and outcome measures, making direct comparisons difficult and reducing our ability to draw definitive conclusions. With regard to the NOS [35], the risk of bias assessment revealed variability in the methodological quality of the included cohort studies, with several studies demonstrating a moderate to high risk of bias. This highlights the need for improved study designs and more rigorous methodologies in future research in order to ensure a more accurate set of results.

The most common dental anomalies reported in the KSA included hypodontia, hyperdontia, microdontia, and impacted teeth. The prevalence of dental anomalies varied widely between the different studies, which may be attributable to differences in study design, sample size, diagnostic criteria, and population characteristics [1,3,4,28,29,30,31,32,33,38]. Some studies reported sexual differences in the prevalence of dental anomalies, indicating that they were more prevalent in women than in men [1,33]; however, other reports showed the opposite, where males were more commonly affected [28,29,30]. In a study conducted on orthodontic patients, most dental anomalies affected the Class I molar relationship [38].

Our findings demonstrate that dental anomalies may affect the number, size, shape, or structure of the affected tooth or teeth. A combination of one or more anomalies is not rare and has been previously reported by Alshukairi (2021) [53]. In most of the included studies, it was difficult to determine the ethnicity or nationality of the participants, as the majority did not include this information. Bakhurji et al. (2021) reported that the majority of their participants were Saudi (*n* = 1540, 81.2%), with non-Saudis accounting for 18.8% (*n* = 357) of their examined sample [31].

Dental anomalies can lead to abnormalities in arch length and occlusion, subsequently affecting the proposed orthodontic treatment plan [3]. These visible changes can affect the overall aesthetics of patients and, in certain cases, their quality of life [25]. Most developmental anomalies were reported to be in the mixed dentition stage [29,31,33], as reflected in various studies. However, many studies included a wide age range to reflect their sample characteristics. This shows that there is a delay in diagnosing dental anomalies, along with a need for earlier diagnosis and intervention, which may necessitate changes in the health system and dental guidelines. As mentioned above, most of the dental anomalies were diagnosed in the mixed dentition stage, usually affecting permanent teeth. Nevertheless, one study demonstrated that none of the participants showed any anomalies in the primary dentition stage [33]. Some of the reviewed studies included adult patients (age range: 18–40 years) (e.g., Vani et al., 2016) [28].

The results of this review have important implications for clinical practice, policymaking, and future research. For practice, these findings suggest a need for more standardized protocols or guidelines to ensure consistent and high-quality care, especially in areas where evidence is either limited or variable. The results highlight gaps in the current evidence base, underscoring the need for well-designed, large-scale studies that can provide stronger data to confirm or challenge existing assumptions. 

## 5. Conclusions

This study provides a comprehensive overview of the prevalence and types of dental anomalies reported in different regions of the Kingdom of Saudi Arabia. The prevalence of dental anomalies in the reported studies ranged from 2.6% to 45.1%, regardless of the number of anomalies assessed [1,3,28,29,30,33].

Our findings highlight the importance of early detection and correct intervention for dental anomalies in order to ensure better overall dental development and aesthetics. Dental practitioners should be made aware of the prevalence and types of dental anomalies in their region, enabling them to provide tailored care and more comprehensive treatment plans for their patients. Further research will be required in order to assess the etiology and genetic basis of dental anomalies in the KSA, as well as to establish more standardized diagnostic criteria and methodologies for future studies.

## Figures and Tables

**Table 1:** Diagram illustrating the risk of bias assessment for the cohort studies included in this review using the Newcastle–Ottawa Scale.

Study	Selection				Comparability	Outcome			
	Representativeness of the Exposed Cohort	Selection of Non-Exposed Cohort	Ascertainment of Exposure	Outcome of Interest Not Present at Start of Study	Comparability of Cohorts	Ascertainment of Outcome	Was Follow-Up Long Enough for Outcomes to Occur?	Adequacy of Follow-Up of Cohorts	Total Score
Ghaznawi, H.I.; Daas, H.; Salako, N.O. (1999) [4]	1	0	1	1	0	1	0	0	4
Afify, A.R.; Zawawi, K.H. (2012) [3]	1	0	1	1	0	1	0	0	4
Vani, N.V.; Saleh, S.M.; Tubaigy, F.M.; Idris, A.M. (2016) [28]	1	0	1	1	0	1	0	0	4
Yassin, S. (2016) [29]	1	0	1	0	0	1	0	0	3
Aljuaid et al. (2022) [30]	0	0	1	0	0	1	0	0	2
ALHumaid et al. (2021) [1]	0	0	1	0	0	1	0	0	2
Bakhurji et al. (2021) [31]	0	0	1	0	0	1	0	0	2
Qutub et al. (2021) [32]	0	0	1	0	0	1	0	0	2
Renugalakshmi et al. (2023) [33]	0	0	1	0	0	1	0	0	2
Al-Jabaa and Aldrees (2013) [38]	0	0	1	0	0	1	0	0	2

**Table 2:** Characteristics of the included studies that reported the prevalence of dental anomalies in Saudi Arabia (M: males; F: females; -: not available).

Study		Ghaznawi et al. (1999) [4]	Afify, A.R.; Zawawi, K.H. (2012) ) [3]	Al-Jabaa and Aldrees (2013) [38]	Vani et al. (2016) [28]	Yassin, S. (2016) [29]	Aljuaid et al. (2022) [30]	ALHumaid et al. (2021) [1]	Bakhurji et al. (2021) [31]	Qutub et al. (2021) [32]	Renugalakshmi et al. (2023) [33]
City/Region		Jeddah	Western region—Jeddah	-	Jazan	Abha	Taif	Eastern Province	Eastern	Jeddah	
Number of Participants		1010	878	602	1000	1252	2481	1104	1897	2045	1442
Males		532 (52.7%)	-	-	500 (50%)	638 (50.9%)	1444 (26.86%)	455 (41.2%)	(52.6%)	-	690
Female		478 (47.3%)	-	-	500 (50%)	614(49.1%)	1037 (41.79%)	649 (58.8%)	(47.4%)	-	792
Prevalence of Anomalies	No. (%)	396 (45.1%)	-	378 (37.8%)	318 (25.79%)	512 (20.63%)	401 (36.3%)	-	-	-	-
	M	-	-	192 (38.4%)	175 (26.95%)	324 (63.4%)	133 (33.2%)	-	-	-	-
	F	-	-	186 (37.2%)	143 (23.28%)	188 (36.6%)	268 (66.8%)	-	-	90 (55.9%)	
Age (Range) in Years		12–40	12–30	-	18–40	5–12	-	7–65	6–18	6–9	5–17
Total Number of Anomalies		-	-	-	-	-	-	993	-	-	-

**Table 3:** The reported dental anomalies in the included studies from Saudi Arabia.

Anomaly Classification	Type/Study	Ghaznawi et al. (1999) [4]	Afify, A.R.; Zawawi, K.H. (2012) [3]	Al-Jabaa and Aldrees (2013) [38]	Vani et al. (2016) [28]	Yassin, S. (2016) [29]	Aljuaid et al. (2022) [30]	ALHumaid et al. (2021) [1]	Bakhurji et al. (2021) [31]	Renugalakshmi et al. (2023) [33]
Number of Anomalies	Missing teeth	-	226 (25.7%)	-	-	121 (9.7%)	-	246 (24.7%)	5.4%	-
	Supernumerary	12(1.19%)	3 (0.3%)	-	10 (1.0%)	44 (3.5%)	80 (15.6%)	1.8%	0.5%	18 (11.18%)
Anomalies in Structure	Amelogenesis imperfecta	-	-	-	-	(0.3%)	-	-	-	-
	Dentinogenesis imperfecta	-	-	-	-	(0.1%)	-	-	-	-
Position Anomalies	Ectopic eruption	-	-	-	76 (7.6%)	(2.3%)	94 (18.3%)	(0.6%)	6%	-
	Positional anomalies	-	-	-	-	-	220 (42.9%)	-	-	-
	Rotation	-	-	-	202 (20.2%)	(1.6%)	126 (24.6%)	(11%)	24.5%	-
Anomalies in Shape	Talon cusp	-	-	-	15 (1.5%)	(1.4%)	28 (5.4%)	(0.1%)	-	-
	Taurodontism	87 (8.61%)	1 (0.1%)	-	29 (2.9%)	(1.4%)	10 (1.9%)	(0.1%)	-	18 (11.18%)
	Fusion	-	-	-	-	(0.8%)	-	-	0.1%	-
	Gemination	-	-	-	-	-	-	-	0.3%	-
	Macrodontia	5 (0.50%)	-	-	6 (0.6%)	(1.8%)	50 (9.7%)	-	-	-
	Microdontia	54 (5.35%)	-	-	9 (0.9%)	33(2.6%)	78 (15.2%)	(1.9%)	-	-
Other Anomalies	Bifid roots	-	-	-	-	-	-	(0.6%)	-	-
	Concrescence/fusion	-	-	-	-	-	17 (3.3%)	(0.1%)	-	-
	Dens invaginatus and dens evaginatus	Invagination 9 (0.89%)	-	-	-	-	11 (2.1%)	(0.2%)	-	-
	Dilaceration	12 (1.19%)	10 (1.1%)	-	72 (7.2%)	-	13 (2.5%)	300 (30.2%)	-	77 (47.83%)
	Ectopic enamel	-	-	-	-	-	-	(1.4%)	-	-
	Fused roots	-	-	-	-	-	-	(17.7%)	-	-
	Hypercementosis	-	-	-	-	-	-	(6.8%)	-	-
	Hypodontia	95 (9.41%)	-	-	52 (5.2%)	-	58 (11.3%)	(0.6%)	-	51 (31.68%)
	Impaction	-	186 (21.1%)	-	-	-	-	-	-	-
	Infraocclusion	-	-	-	-	-	-	-	-	3 (1.86%)
	Odontoma (Not a dental anomaly)	7 (0. 69%)	1 (0.1%)	-	-	-	-	-	-	-
	Peg-shaped	-	-	-	-	-	-	(1.2%)	1.1%	7 (4.35%)
	Retained	-	-	-	-	-				
	Submerged	-	-	-	-	-	-	-	-	-
	Supernumerary roots	-	-	-	7 (0.7%)	-	33(6.4%)	-	-	-
	Transposition	2 (0.20%)	-	-	3 (0.3%)	-	-	3 (0.3%)	-	-
	Transmigration	-	-	-	-	-	-	-	-	-
	Diastema	45 (4.46%)	-	-	-	-	-	-	-	-

## Data Availability

No new data were created or analyzed in this study.

## Supplementary Materials

The following supporting information can be downloaded at: https://www.mdpi.com/article/10.3390/healthcare12232323/s1, Table S1: PRISMA Checklist.
